# Owner‐Directed Canine Aggression in Thailand: Triggers, Associated Factors and Clinical Interventions

**DOI:** 10.1155/vmi/6224941

**Published:** 2026-07-31

**Authors:** Jarawee Supanta, Worakan Boonhoh, Orachun Hayakijkosol, Tuempong Wongtawan

**Affiliations:** ^1^ Akkhraratchakumari Veterinary College, Walailak University, Nakhon Si Thammarat 80160, Thailand, wu.ac.th; ^2^ One Health Research Center, Walailak University, Nakhon Si Thammarat 80160, Thailand, wu.ac.th; ^3^ Veterinary Preclinical Science, College of Science and Engineering, James Cook University, Townsville Queensland, 4811, Australia, health.qld.gov.au; ^4^ Pet Mental and Behaviour Clinic, Walailak University’s Animal Hospital, Nakhon Si Thammarat 80160, Thailand

**Keywords:** behavioural intervention, canine behaviour, feeding toy, fluoxetine, owner-directed aggression

## Abstract

Dog ownership provides physical and psychological benefits; however, inappropriate handling may lead to behavioural problems, particularly owner‐directed aggression, which can compromise human wellbeing. This study investigated factors associated with owner‐directed aggression and evaluated the effectiveness of interventions in Thailand. A randomised, single‐blind clinical trial was conducted in 80 dogs exhibiting moderate to severe owner‐directed aggression (including a history of biting). Aggression was assessed using owner questionnaires and veterinary interviews. Dogs were allocated to four groups: feeding toy (TOY), fluoxetine (DRUG), combination therapy (DRUG + TOY) and control. All groups received behavioural modification guidance. Aggression scores were measured before and after four weeks using a standardised scale. Multivariable analysis indicated that human–dog interactions and management practices were significantly associated with aggression scores, whereas owner and dog demographic factors were not. Baseline aggression did not differ among groups (*p* ≥ 0.05). Following treatment, all groups showed significant improvement (*p* < 0.05), with the DRUG and DRUG + TOY groups demonstrating greater reductions than the TOY and control groups. No significant differences were observed between DRUG and DRUG + TOY or between TOY and control. Mild side effects of fluoxetine, primarily drowsiness and reduced appetite, were observed during the first week and were less frequent in the combination group. Overall, fluoxetine (0.5–1 mg/kg) was more effective in reducing owner‐directed aggression than environmental enrichment and owner education alone. However, enrichment and education play a crucial role in improving dog welfare and in sustainably reducing or preventing aggression and may enhance treatment outcomes when used alongside pharmacological intervention.

## 1. Introduction

Pet ownership, particularly of dogs and cats, offers considerable mental and physical health advantages for individuals. Nonetheless, without appropriate management, it can also pose risks to human health, such as injuries, and contribute to environmental concerns, including pet waste and threats to wildlife [1–5]. A significant issue is aggressive behaviour, such as biting, which jeopardises human safety through trauma and infection and weakens the human–animal relationship [6–8]. These circumstances may lead to pet relinquishment or abandonment, contributing to the rise in stray dog populations and subsequently creating broader public health (e.g., zoonoses and road accidents) and animal welfare challenges [9, 10].

Canine aggression is a multifactorial behavioural issue shaped by the interaction between dog‐related characteristics, owner factors and the surrounding environment. In some cases, underlying medical conditions may contribute to aggressive behaviour, including infections, hormonal imbalances, pain or inflammatory processes [11, 12]. Although certain breeds, such as Chihuahua, Pomeranian, Chow Chow and German Shepherd, have been reported to exhibit a higher prevalence of aggressive tendencies [13–15], breed alone is not a primary or deterministic factor. Rather, behavioural outcomes are influenced by a combination of genetic predisposition and environmental inputs. Early life experiences, such as being raised as the only dog, exposure to inappropriate training practices (e.g., punishment‐based methods) and handling (e.g., excessive or inconsistent petting), may further increase the risk of aggression [13, 16, 17].

Owner‐directed aggression is a serious behavioural issue that can result in biting injuries, commonly affecting the head and hands of adults and the face of children [18, 19]. Children are particularly at risk, as they often misinterpret canine body language, unintentionally provoke discomfort in dogs and exhibit sudden, unpredictable movements [19–21]. Besides physical trauma, dog bites can also transmit zoonotic diseases, including rabies and various bacterial infections [19, 22]. Additionally, such incidents may lead to psychological consequences, including posttraumatic stress disorder (PTSD), cynophobia and long‐term emotional distress associated with scarring [23].

Management of canine aggression typically involves behaviour modification, environmental adjustments and, when necessary, pharmacological treatment [24]. Owner‐directed aggression is commonly treated with selective serotonin reuptake inhibitors (SSRIs), particularly fluoxetine [24–26]. However, concerns arise due to potential side effects, including anorexia, depression, ataxia, vomiting, tachycardia and tachypnoea [27–29]. In Thailand, behavioural medicine is rarely practised, and research in this area is lacking, despite the suspected high prevalence of dog aggression [1]. This study aimed to identify factors associated with owner‐directed aggression and to evaluate the effectiveness of toys and fluoxetine in managing this behaviour in dogs.

## 2. Material and Methods

### 2.1. Ethical Approval

This project was approved by the Walailak University Institutional Animal Care and Use Committee (WU‐ACUC) (ID: 67082; approved date: 31 Dec 2024) and the Human Research Ethics Committee (WUEC) (ID: 24‐382‐01; approved date: 6 Nov 2024).

### 2.2. Recruitment Process

Eighty dogs with a history of biting their owners were invited to participate in the project at the Walailak University’s Animal Hospital. Only one dog per household was eligible for study. Inclusion criteria were owners aged over 20 years, dogs aged over 1 year and dogs displaying moderate to severe owner‐directed aggression. Concurrent disease and treatment were recorded; however, eligible dogs were required to have previously undergone nonpharmacological interventions without success and must not have been exposed to feeding toys, psychotropic medications (e.g., fluoxetine) or behavioural‐modifying supplements (e.g., GABA, tryptophan, CBD or L‐theanine).

Exclusion criteria were dogs that became seriously ill during treatment, owners unable to follow the study guidelines or owners who failed to report the behaviour change or attend scheduled meetings.

### 2.3. Diagnosis of Owner‐Directed Aggression

The diagnosis involved a physical examination and blood testing to rule out any medical conditions that could contribute to behavioural problems. Owners were requested to complete an online questionnaire, developed using Google Forms (Google LLC, CA, USA), to collect demographic information regarding themselves, their dogs, the home environment and their interactions. This was completed prior to a detailed interview with a veterinary behaviourist, who assessed and confirmed owner‐directed aggression and identified its potential type and underlying cause. All owners also provided video recordings demonstrating their dogs’ aggressive behaviour. In this study, aggression in dogs is defined as repeated behaviour intended to threaten, intimidate or harm a person or another animal, including actions such as threatening growling or barking, baring teeth, intentional stiffening, lunging, snapping, biting or attempting to bite.

### 2.4. Type of Canine Owner‐Directed Aggression

In this study, owner‐directed aggression was divided into five subgroups, as shown in Table 1. Fear‐induced aggression refers to aggression shown when a dog feels threatened, such as when the owner stares at, scolds or verbally reprimands the dog or appears likely to strike it. Possessive aggression occurs when the owner removes toys, food or valued items (e.g., dolls, bowls) from the dog or approaches the dog while it is eating or playing with a toy. Grooming‐induced aggression is shown during activities such as bathing, nail trimming or brushing carried out by the owner. Territorial aggression is characterised by dogs showing aggressive behaviour when their owner steps over them or walks very close to them while they are resting. Petting‐induced aggression describes situations where aggression arises when the owner pets, hugs, holds, sniffs or kisses the dog.

**TABLE 1:** Types of dog aggression and trigger events.

Types of aggression	Trigger events/behavioural tests
Fear‐induced aggression	Occurs when the dog perceives the owner’s actions as threatening, such as direct staring, scolding, shouting, sudden harsh movements or when the owner raises a hand or object in a way that may appear threatening. May be triggered during owner approach when the dog feels cornered or unable to escape.
Possessive (resource‐guarding) aggression	Triggered when the owner attempts to take away or handle valued resources, such as food, treats, toys, bones or personal objects the dog has claimed. Also triggered when the owner approaches the dog while it is eating, chewing or playing.
Grooming‐ or handling‐induced aggression	Occurs during routine handling or husbandry tasks, such as bathing, brushing, drying, ear cleaning, tooth brushing or nail trimming. May also appear during lifting, putting on harnesses or wiping paws.
Territorial or space‐related aggression	Triggered when the owner enters or intrudes upon the dog’s personal space, such as stepping over the dog, walking closely past the dog while it is resting or attempting to move the dog from a resting or sleeping area. May also occur when the dog guards a particular location (sofa, bed, doorway).
Petting‐ or contact‐induced aggression	Occurs when the owner touches or physically interacts with the dog, such as petting, hugging, holding, kissing or handling sensitive areas (head, paws, tail). May also occur during prolonged or unwanted contact, even when initiated gently.

### 2.5. Aggression Score of Canine Owner‐Directed Aggression

In this study, an aggression score was developed to quantitatively assess treatment effects. The canine owner‐directed aggression score was calculated as the mean of the aggressive behaviour ratings (0–4) across five triggering events (Table 2). For each event, owners rated their dog’s aggression from 0 (no aggression) to 4 (very aggressive, such as frequent biting). This questionnaire had been previously tested in a pilot study, and reliability analysis demonstrated excellent internal consistency (McDonald’s *ω* = 0.91). Principal component analysis revealed that all five aggression types loaded onto one component, with loading values ranging from moderate to high (0.487–0.722). These results justify the use of the mean aggression score as a valid indicator for assessing treatment outcomes. Owners completed the aggression ratings for all five triggering events both before and after treatment. To minimise reporting bias, follow‐up interviews were conducted to verify owner‐reported responses, and standardised scoring criteria were provided to improve consistency across participants.

**TABLE 2:** Scale of dog aggressive behaviour rated by their owners.

Aggressive behaviour scale	Short description	Description of aggressive behaviour
0	None	No aggressive behaviour observed. The dog has never shown aggressive behaviours such as growling, baring teeth, snapping or biting.
1	Rare mild aggression	Rarely observed mild aggressive behaviours such as slight growling, barking or baring teeth
2	Occasional mild aggression	Aggressive behaviours appear occasionally but are low in intensity, with signs such as growling, barking, stiffening or baring teeth occurring only in specific situations or triggers, and without any episodes of snapping or biting.
3	Moderate aggression	The dog frequently shows behaviours such as growling, barking, stiffening or baring teeth and also occasionally exhibits more severe aggression, including snapping, biting or attempts to bite.
4	Very aggressive	Severe aggressive behaviours occur frequently, including regular snapping, biting, repeated attempts to bite or attacks and may happen with or without warning.

*Note:* This scale was rated in each event that may trigger aggressive behaviour in dogs.

### 2.6. Experimental Design

**FIGURE 1:**
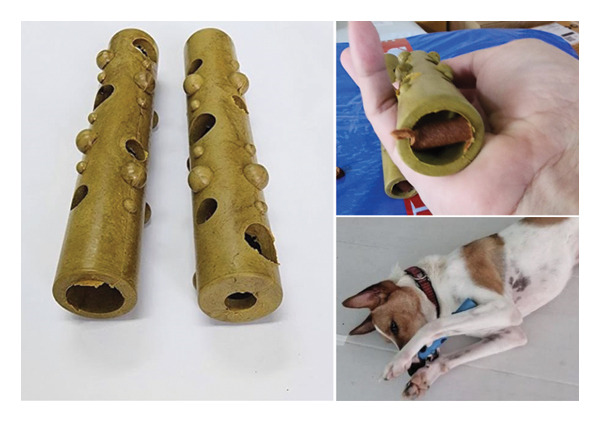
Feeding toys made by para‐rubber.

Due to the nature of the behavioural intervention involving a feeding toy, complete blinding was not feasible, as owners were aware of whether their dogs received the toy. However, to minimise assessment bias, the veterinarian responsible for evaluating behavioural outcomes was blinded to the treatment allocation, except when behavioural management instructions were required. Therefore, the study employed a partially single‐blind design, allowing for partial blinding of assessors while acknowledging the practical limitations of fully blinding participants. Aggressive dogs were assigned to four treatment groups. The first group was provided with a para‐rubber feeding toy (TOY), designed and manufactured by Walailak University [30] (Figure 1), along with a multivitamin placebo. Dogs in this group interacted with the toy twice daily, with each session lasting at least 30 min. The second group received fluoxetine (GPO, Bangkok, Thailand) at a dosage of 0.5–1 mg/kg once daily (DRUG). The third group was assigned both treatments (DRUG + TOY). The fourth group functioned as the control group and was given neither the feeding toy nor the medication but instead received a placebo drug. Treatments were administered for a minimum duration of 4 weeks. Comprehensive behavioural assessments, including owner interviews, questionnaires and aggression score evaluations, were performed at baseline and after 4 weeks of treatment. Owners also reported daily medication adherence and brief behavioural observations via the LINE online application (LY Corporation, Tokyo, Japan). Dogs that continued to exhibit residual aggressive behaviours (e.g., occasional biting) after 4 weeks—despite reductions in overall aggression scores and attack frequency—remained on the same treatment regimen until behavioural resolution was achieved. These cases generally involved dogs with high baseline aggression scores.

### 2.7. Sample Size Calculation

The sample size was calculated for a before–after study using a paired *t*‐test (matched‐pairs design), based on the expected mean difference in aggression scores before and after intervention, following standard methods [31]. The calculation used

(1) N=Z12−α/+Z1−β×S∆E2,

where *α* = 0.05 and power = 80%. Parameters were derived from a previous study [32], yielding a required sample size of *N* = 45. Given the inclusion of four treatment groups and the use of nonparametric analyses, the sample size was increased to 20 dogs per group (*N* = 80) to ensure adequate power, as nonparametric tests typically require larger samples [33]. An additional allowance was made for an anticipated attrition rate of 25%–30%.

## 3. Statistical Analysis

Statistical analyses were conducted using Jamovi software (Version 2.6.26) [34]. A *p* value < 0.05 was considered statistically significant. As the data were nonparametric, the Wilcoxon signed‐rank test was used to compare differences before and after treatment. The Kruskal–Wallis (KW) test followed by Dwass–Steel–Critchlow–Fligner (DSCF) pairwise comparisons were applied to assess differences among the four treatment groups. Spearman’s rank correlation was used to examine relationships between aggression types, and Fisher’s exact test was used to compare treatment side effects.

Associations between demographic variables and aggression scores were initially explored using univariable analyses, including the Mann–Whitney *U* test and KW test with DSCF post hoc comparisons, as appropriate. Subsequently, a generalised linear model (GLM) was used to evaluate the association between aggression score and multiple owner‐related factors while controlling for potential confounders. Variables with *p* < 0.20 in univariable analyses were included in the multivariable GLM. Final model selection was based on statistical significance and biological relevance.

## 4. Results

### 4.1. Demographics

The details of demographic data are shown in Table S1. The study included 80 dog owners (detailed in Table S1), the majority of whom were women (83.75%). Participants ranged widely in age, with the largest proportion aged 41–50 years (31.25%), followed by those aged 31–40 years (26.25%) and 51–60 years (22.50%); smaller proportions were aged 20–30 years (11.25%) or over 60 years (8.75%). Most owners reported substantial experience in dog ownership, with 60% having more than five years of experience, while the remaining had five years or less.

Of the 80 dogs, most were male (63.75%) and purebred (71.25%), with Chihuahua being the most common pure breed. Most dogs were young adults, with 71.25% aged 1–4 years, followed by 23.75% aged 4–8 years and only 5% over 9 years of age. Regarding reproductive status and size, more than half of the dogs were neutered (56.25%) and in medium to large size (55.00%).

Most dogs resided in undetached houses (92.50%) and were primarily kept indoors (60.00%), followed by semi‐indoor environments (30.00%) and cages (10.00%). Only 26.25% were frequently restrained by chaining. The majority of households consisted of 2–4 adults (68.75%), and 25.00% included children under 18 years of age. Nearly half of the households owned a single dog (48.75%), while 25.00% kept two dogs and 26.25% had more than two. Additionally, 30.00% of households kept other animal species, with cats being the most common (58.33%, *n* = 14/24). Over half of the dogs (52.50%) had lived in their current environment for more than 24 months.

The majority of dogs were fed commercial diets (73.75%) and received meals twice daily (67.50%), while 20.00% were fed either three times daily or allowed free access to food, and 12.50% were fed only once per day. Most dogs exhibited an ideal body condition score (BCS 4–6; 61.25%), whereas 12.50% were underweight and 26.25% were overweight. Chronic health problems were reported in 20.00% of dogs, with skin disorders being the most common (43.75%, *n* = 7/16). In terms of exercise, 38.75% of dogs were walked for 15–30 min daily, 32.50% for less than 15 min, 12.50% for 30–60 min, and 16.25% had no regular exercise routine.

Human–dog interactions were predominantly positive. Nearly all dogs (96.25%) received positive reinforcement, whereas 36.25% were frequently subjected to punishment‐based training methods. Furthermore, the majority of dogs experienced frequent affectionate behaviours from their owners, including hugging (85.00%), head petting (85.00%), body petting (90.00%) and kissing (81.25%). Walking was a common activity (63.75%), whereas travelling with dogs was less frequently reported (47.50%). Play using nonfeeding toys was prevalent, occurring in 70.00% of dogs. In contrast, the provision of artificial bones showed a nearly equal distribution, with 47.50% of owners offering them often and 52.50% offering them infrequently. A substantial proportion of dogs did not receive scent enrichment (81.25%), auditory stimulation through music (68.75%) or visual enrichment such as television exposure (53.75%). Basic obedience training was routinely implemented in 61.25% of the dogs. Sleeping arrangements were almost evenly distributed, with 48.75% of dogs sharing the same room as their owners and 51.25% sleeping in a separate area. Additionally, more than half of the dogs (55.00%) were left unattended for periods exceeding 9 hours per day.

### 4.2. Occurrence of Owner‐Directed Aggression Trigger Type

Among the dogs assessed, fear‐triggered (induced) aggression was the most prevalent trigger, observed in 68 dogs (85.00%) with a median behaviour score of 3.50 (IQR = 1.00). Possessive‐triggered aggression occurred in 55 dogs (68.80%) and grooming‐triggered aggression in 43 dogs (53.8%), both with a median score of 3.0 (IQR = 2.00), indicating moderate occurrence and intensity. In contrast, territorial‐triggered aggression and petting‐triggered aggression were less common, affecting 17 (21.30%) and 18 dogs (22.50%), respectively, both with lower median scores of 1.0 (IQR = 2.00).

### 4.3. Co‐occurrence and Correlation Among Types of Owner‐Directed Aggression

Co‐occurrence of aggression subtypes was identified in the majority of dogs (80%). The most common pattern involved two concurrent aggression types (35%), followed by three types (25%) and a single type (20%). Smaller proportions of dogs exhibited four types (11.25%) or all five aggression types (8.75%).

Spearman correlation analysis (Table 3) identified several significant relationships among the aggression subtypes. Possessive aggression showed a positive correlation with fear‐related aggression (*ρ* = 0.28, *p* = 0.01). Territorial aggression was similarly associated with fear‐related aggression (*ρ* = 0.23, *p* = 0.04). Petting‐related aggression was positively correlated with both possessive aggression (*ρ* = 0.23, *p* = 0.04) and territorial aggression (*ρ* = 0.41, *p* < 0.001).

**TABLE 3:** Correlation between types of dog aggression.

		Fear	Possessive	Groom	Territory	Petting
Fear	*ρ*	—				
	df	—				
	*p* value	—				

Possessive	*ρ*	0.28	—			
	df	78	—			
	*p* value	0.01^∗^	—			

Groom	*ρ*	0.19	0.20	—		
	df	78	78	—		
	*p* value	0.08	0.06	—		

Territory	*ρ*	0.23	0.20	0.15	—	
	df	78	78	78	—	
	*p* value	0.04^∗^	0.07	0.16	—	

Petting	*ρ*	0.04	0.23	0.18	0.41	—
	df	78	78	78	78	—
	*p* value	0.73	0.04^∗^	0.09	0.0001^∗∗^	—

*Note:* ρ = Spearman’s rho.

^∗^ *p* < 0.05.

^∗∗^ *p* < 0.001.

### 4.4. Association Between Demographic Factors and Aggression Scores (Univariable Analysis)

The detailed statistical associations between demographic factors and aggression scores are presented in Tables S1, S2 and S3. Most demographic factors showed no significant association with aggression (*p* ≥ 0.05), except human–dog interaction factors, including head petting and travelling with the dog (*p* < 0.05). Dogs whose owners engaged in head petting less frequently exhibited significantly higher overall aggression scores (median = 2.7) compared with dogs whose owners often performed head petting (median = 2.00) (*U* = 247, *p* = 0.03, *r* = 0.39). Dogs that frequently travelled with their owners displayed significantly (*U* = 573, *p* = 0.03, *r* = 0.28) higher aggression scores (median = 2.50) than dogs that rarely travelled with their owners. Additionally, regular provision of artificial bones and food type showed a marginally significant negative association with aggression (*p* = 0.05). Dogs receiving artificial bones frequently exhibited lower aggression scores (median = 2.00) compared with those that rarely or never received them (median = 2.50). Dogs fed home‐cooked diets tended to exhibit higher aggression scores (median = 2.40) compared with those fed commercial diets (median = 2.00).

Specifically, head petting was associated with pet‐induced aggression (*U* = 218, *p* = 0.008, *r* = 0.46) and territorial aggression (*U* = 221, *p* = 0.009, *r* = 0.45), while travelling with dogs was associated with grooming‐induced aggression (*U* = 454, *p* = 0.0007, *r* = 0.43).

### 4.5. Multivariable Analysis of Independent Factors Associated With Aggression

Variables were selected for the multivariable GLM based on univariable screening (*p* < 0.20) and biological relevance. These included owner age, food type, living place, head petting, travelling with the dog and provision of artificial bones, as they represent key factors including owners, dogs, environment and human–dog interaction. To avoid overfitting and multicollinearity, only representative variables were retained in the final model.

The model showed a significant overall fit (*F* = 2.90, *p* = 0.003), explaining 34.2% of the variance in aggression scores (*R* ^2^ = 0.342). Several factors were significantly associated with aggression scores, indicated as independent predictors for aggression. These predictors included head petting (*F* = 4.84, df = 1, *p* = 0.03), travelling with the dog (*F* = 7.85, df = 1, *p* < 0.01), provision of artificial bones (*F* = 4.80, df = 1, *p* = 0.03) and living area (*F* = 3.50, df = 2, *p* = 0.04). In contrast, food type (*F* = 3.01, df = 1, *p* = 0.09), owner age (*p* = 0.71) and breed type (purebred vs mixed; *p* = 0.47) were not significantly associated with aggression. These findings suggest that management practices and human–dog interactions, rather than owner and dog characteristics, play a more prominent role in influencing aggressive behaviour.

### 4.6. Effect of the Treatments on Owner‐Directed Aggression

Of the 80 dogs initially recruited, several were excluded from the study due to owners’ noncompliance with the 4–8‐week protocol, such as irregular medication administration, failure to provide daily behavioural reports or failure to attend scheduled veterinarian appointments. The numbers of dogs remaining in each group are shown in Table 4: 14 in the control group, 13 in the TOY group, 15 in the DRUG group and 15 in the DRUG + TOY group.

**TABLE 4:** Comparison of owner‐directed aggression scores before and after treatment.

Groups	N	Median of aggression score (IQR)		Statistic (W)	*p* value	Median difference	Effect size
		Before	After				
Control	14	2.70 (1.00)	2.50 (1.00)^a^	105.00	0.003^∗^	−0.20	1
TOY	13	2.78 (0.97)	2.00 (0.89)^a^	91.00	0.002^∗^	−0.78	1
DRUG	15	2.78 (0.55)	0.80 (0.75)^a^	120.00	0.0007^∗^	−1.98	1
DRUG + TOY	15	2.80 (1.17)	1.00 (0.33)^a^	120.00	< 0.0001^∗^	−1.80	1
Statistics (H)		0.24	26.72				
*p* value		0.96	< 0.0001^#^				

*Note:* Effect size used rank biserial correlation.

^∗^Statistically significant difference (*p* < 0.05) between before and after treatment (row) by the Wilcoxon signed‐rank test (W).

^#^Statistically significant difference (*p* < 0.05) among study groups (column) by the Kruskal–Wallis test (H).

^a^Statistically significant difference (*p* < 0.05) between groups by the multiple comparison using Dwass–Steel–Critchlow–Fligner test (DSCF).

The treatment effects are summarised in Table 4. At baseline (prior to treatment), no statistically significant differences (*p* ≥ 0.05) were observed in owner‐directed aggression among the groups. Following treatment, significant reductions (*p* < 0.05) were observed across all groups, including the control group. As all owners received an education on canine behaviour and recommendations for behaviour modification, this shared educational component likely contributed to the small change, yet statistically significant reduction seen in the control group (median difference −0.20).

The TOY group showed a greater reduction in aggression scores (−0.78), suggesting that feeding toy enrichment provided an additional benefit beyond education alone; however, this reduction did not reach statistical significance (*p* ≥ 0.05).

Notably, DRUG‐based interventions (with or without TOY) were significantly more effective (*p* < 0.05) than both the control and TOY‐only conditions. The DRUG group exhibited the most pronounced improvement (−1.98), while the DRUG + TOY group also demonstrated a substantial reduction (−1.80), with no statistical difference between them. These findings suggest that pharmacological therapy (fluoxetine), either alone or in combination with enrichment (feeding toy), was the most effective treatment strategy.

### 4.7. Side Effects of Treatment

No side effects were observed in dogs treated with toys alone. The most common side effects of fluoxetine treatment during the first week were sleepiness (90%, *n* = 18/20) and mild anorexia (60%, *n* = 12/20). In dogs receiving the combination of fluoxetine and a toy, side effects appeared slightly lower, with sleepiness reported in 75% of dogs (*n* = 15/20) and mild anorexia in 50% (*n* = 10/20). However, there was no statistically significant difference in side effects between these two groups (*p* ≥ 0.05). The side effects were found only the first week of the treatment.

## 5. Discussion

### 5.1. Significant Factors Associated With Owner‐Directed Aggression

In this study, management practices and human–dog interactions were identified as key factors associated with owner‐directed aggression, whereas owner characteristics were not significantly associated. Specifically, food type, head petting, travelling, provision of artificial bones and living area were significantly associated with aggression scores in the multivariable model. Human–dog interaction factors also played a prominent role. Dogs that experienced less frequent head petting showed significantly higher aggression scores, while frequent head petting was associated with lower aggression. This may indicate that appropriate physical interaction contributes to positive social bonding and behavioural stability. However, it is also possible that owners of aggressive dogs avoid physical contact, highlighting the potential for reverse causation. Previous studies have reported that intrusive or poorly timed handling can trigger aggression, particularly in dogs with low tolerance to physical contact [35, 36], suggesting that both the quality and context of interaction are critical.

Similarly, frequent travelling was associated with increased aggression scores. Travel may expose dogs to unfamiliar, unpredictable or stressful environments, thereby lowering their threshold for aggressive responses. This is consistent with previous findings indicating that fear, stress and aversive handling experiences contribute to aggression [37, 38]. Dogs are often transported for veterinary visits or grooming procedures, which are commonly perceived as negative experiences, and may, therefore, develop anticipatory stress or defensive behaviour. Prevention of aggression and stress associated with travelling is essential. This may be achieved through improved owner education and appropriate behavioural interventions. In situations where travel is unavoidable, strategies such as positive reinforcement training and, where necessary, the use of pharmacological support may help to reduce stress before and after travel.

Dogs fed home‐cooked diets showed higher aggression scores than those fed commercial diets, likely reflecting greater nutritional variability. Diet influences neurochemical pathways—particularly serotonin, which regulates mood and impulse control [39, 40]. Imbalances in key nutrients from home‐prepared diets may therefore contribute to aggression [41]. For instance, insufficient tryptophan can reduce serotonin synthesis [40, 42], while high‐protein diets may disrupt amino acid balance, limiting tryptophan uptake into the brain or increasing tyrosine‐derived excitatory neurotransmitters [41, 43, 44]. Additionally, omega‐3 deficiency may increase inflammation [45] and predispose to physical conditions that heighten irritability and reactivity, thereby contributing to aggression [11].

The provision of artificial bones was associated with lower aggression scores, suggesting a beneficial effect of environmental enrichment, particularly chewing or feeding enrichment. Such enrichment may reduce boredom, frustration and stress by providing an appropriate outlet for natural chewing behaviour, increasing activity levels, enhancing cognitive engagement and supporting physical health, thereby promoting more stable and appropriate behavioural responses [30, 32, 46–48]. In addition, enrichment can lower baseline arousal and redirect potentially destructive behaviours, excessive vocalisation or aggressive tendencies into nonharmful activities [30, 32, 47]. Repetitive chewing behaviour has also been associated with calming effects, which may support emotional regulation and contribute to improved dental health [30, 32, 49, 50]. Taken together, chewing/feeding enrichment can enhance canine welfare and may reduce undesirable behaviours, including aggression [30, 32, 47, 51].

In this study, most owners were middle‐aged females, consistent with previous reports in Thailand [1]. Although owner demographics were not significantly associated with aggression scores, this distribution may reflect caregiving roles and engagement in dog management. The study population was predominantly composed of young adult dogs and small purebred breeds, particularly Chihuahuas, which have been reported to exhibit higher levels of owner‐directed aggression [1, 52–54]. However, no association was found between aggression and breed type (purebred vs mixed) or body size in this study. Similar variability has been reported across countries, with different breeds implicated in aggression in the United States, Australia and China [14, 52, 55, 56]. These findings suggest that breed alone is not a deterministic factor and that aggression likely arises from a complex interaction between genetic, environmental and human–dog interaction factors.

### 5.2. Owner‐Directed Aggression Rose From Multiple Types of Triggers

In the present study, most dogs exhibited more than two types of owner‐directed aggression, with fear‐based and possessive aggression being the most prevalent. Fear‐triggered aggression has consistently been reported as the most common form of dog aggression, including owner‐, stranger‐ and dog‐directed aggression [53, 57, 58]. Although research on the co‐occurrence of aggression subtypes (trigger) in owner‐directed cases is limited [59], our finding that many dogs displayed multiple aggression types supports the view that dog aggression is rarely driven by a single motivation/trigger. Rather, it reflects overlapping emotional states—such as fear, anxiety, possessiveness and territoriality—consistent with the concept of mixed‐motivation/trigger aggression [35]. Additionally, grooming‐induced aggression was newly identified as one of the common aggression types/trigger in this study. Grooming may trigger aggression due to fear, anxiety, discomfort or pain, particularly in dogs with previous negative grooming experiences [37, 60].

### 5.3. Intervention of Owner‐Directed Aggression in Dogs

Regarding treatment, fluoxetine demonstrated the greatest reduction in aggression scores, either alone or in combination with a feeding toy, supporting its established role in managing canine aggression [25, 61, 62]. Although other psychotropic drugs have been used to treat dog aggression, many lack strong evidence of efficacy [63]. The effectiveness of fluoxetine observed at a relatively low dose (0.5–1 mg/kg) suggests that clinically meaningful improvement can be achieved while minimising adverse effects. Feeding toys provided moderate benefits, likely through increased mental stimulation and reduction of boredom‐related stress, but were less effective as a standalone intervention. The small but significant improvement observed in the control group may be attributed to owner education and behavioural guidance, highlighting the importance of nonpharmacological management.

The present study demonstrated that veterinary‐provided education on canine behaviour, combined with guidance on behaviour modification, resulted in a modest but statistically significant reduction in aggression. This suggests that even brief, structured education can positively influence behavioural outcomes, likely through improved owner understanding of canine body language, enabling earlier recognition of stress signals and more appropriate responses, such as the use of positive reinforcement before the escalation of aggressive behaviour [49, 59]. In addition, education may enhance the management of environmental triggers by promoting appropriate living conditions and enrichment (e.g., designated resting areas and toys), more predictable routines and increased physical and mental stimulation, all of which contribute to improved emotional regulation [59, 64].

No adverse effects were observed in dogs treated with the feeding toy alone, supporting the safety of environmental enrichment as a nonpharmacological intervention. In contrast, fluoxetine‐treated dogs commonly exhibited transient side effects during the first week, particularly somnolence and mild anorexia, likely reflecting early neurochemical adaptation [27–29]. Although the combination of fluoxetine and a feeding toy showed a slightly lower frequency of side effects and reduced aggression scores compared with fluoxetine alone, this difference was not statistically significant. Nevertheless, environmental enrichment may help mitigate stress and sedative effects through increased activity and engagement. Importantly, all side effects were mild, self‐limiting and resolved within the first week, consistent with previous reports [27–29].

However, the modest effect size indicates that while education alone is beneficial, it may be insufficient for more severe or chronic cases, particularly those involving complex behavioural or underlying psychological conditions. This highlights the potential need for adjunctive pharmacological interventions. Nevertheless, these findings support the integration of owner‐focused behavioural education into routine veterinary practice, or even prior to pet ownership, as a practical, scalable and sustainable approach to reducing canine aggression and other undesirable behaviours [65, 66].

### 5.4. Small Side Effects of Treatment

No adverse effects were observed in dogs treated with the feeding toy alone, supporting the safety of environmental enrichment as a nonpharmacological intervention. In contrast, dogs receiving fluoxetine commonly exhibited transient side effects during the first week of treatment, particularly somnolence and mild anorexia. These findings are consistent with previous reports as neurochemical adaptation occurs [27–29].

Although the combination of fluoxetine and a feeding toy showed a slightly lower frequency of side effects and aggression score compared with fluoxetine alone, this difference was not statistically significant. Nevertheless, the trend may suggest that environmental enrichment could help play activity result in mitigating stress and sleepiness. Importantly, all observed side effects were mild and limited to the first week of treatment, with no persistent or severe adverse events reported. This transient profile aligns with the existing literature, which indicates that most SSRI‐related side effects in dogs are self‐limiting and tend to resolve as treatment continues [27–29].

Overall, these findings support the clinical safety of fluoxetine for managing owner‐directed aggression, particularly when combined with behavioural and environmental interventions. Veterinarians should, however, inform owners about the possibility of mild, short‐term side effects during the initial treatment period to ensure appropriate expectations and adherence to therapy.

### 5.5. Limitation and Future Direction

This study has several limitations. First, its single‐centre design reflects the limited accessibility of behavioural medicine services in Thailand and may restrict the generalisability of the findings. Second, the relatively short intervention period may not fully capture long‐term treatment outcomes or the risk of relapse. In addition, the study population was highly heterogeneous, encompassing a wide range of ages, breeds and aggression subtypes, which may have influenced treatment responses, as these factors are known to be associated with aggression [14, 52, 55, 56].

Potential bias from owner‐reported questionnaire data should be considered. Although follow‐up interviews and a within‐subject (pre–post) design were used to verify responses and minimise bias, reporting inaccuracies and subjective interpretation cannot be entirely excluded, as owners’ perceptions of aggression may vary with their experience, expectations and tolerance thresholds, even with standardised definitions. Additionally, unmeasured external factors—such as owner behaviour, training consistency and environmental conditions—may have influenced the observed outcomes.

Future research should focus on more homogeneous populations, particularly commonly affected breeds and specific subtypes of owner‐directed aggression, to improve the precision of findings. Studies incorporating longer follow‐up periods are warranted to evaluate sustained treatment efficacy and relapse rates. In addition, comparative investigations of pharmacological and nonpharmacological interventions would help strengthen the evidence base and support the development of more effective, context‐specific clinical guidelines for managing owner‐directed aggression in dogs in Thailand.

## 6. Conclusion

Owner‐directed aggression in dogs is a multifactorial condition, with fear‐related and possessive aggression being the most common and frequently co‐occurring triggers. Aggression was more strongly associated with human–dog interactions and management practices than with owner or dog characteristics. Fluoxetine (0.5–1 mg/kg) was the most effective intervention; however, its combination with environmental enrichment (feeding toys) and owner education may further enhance animal welfare and support more sustainable reductions in aggression. Preventive strategies should emphasise owner education, appropriate handling, environmental enrichment and minimising stress during potentially aversive situations such as travelling. Early modification of these factors may help reduce the development and severity of owner‐directed aggression. Future research should investigate long‐term outcomes, evaluate alternative pharmacological options, optimise interventions for mild to moderate aggression, include larger and more diverse populations and examine the role of owner knowledge in influencing behavioural outcomes.

## Author Contributions

Jarawee Supanta: formal analysis, investigation, writing–original draft and writing–review and editing.

Worakan Boonhoh: resource, investigation, validation and writing–review and editing.

Orachun Hayakijkosol: supervision, validation and writing–review and editing.

Tuempong Wongtawan: conceptualisation, methodology, validation, formal analysis, investigation, resource, writing–original draft, writing–review and editing, funding acquisition and project administration.

## Funding

This project was financially mainly supported by the Fundamental fund (WU‐FF68‐26) of Thailand Science Research and Innovation and partially supported by the Reinventing Project for Enhancing Thai Universities into the International Education, the Ministry of Higher Education, Science, Research, and Innovation, to Tuempong Wongtawan.

## Disclosure

A preliminary version of this manuscript has been published as a preprint and is available online at preprints.org [67]. This earlier version contains less data and fewer analyses than the current manuscript.

## Conflicts of Interest

The authors declare no conflicts of interest.

## Supporting Information

Additional supporting information can be found online in the Supporting Information section.

## Supporting information


**Supporting Information** Table S1: demographic characteristics, Table S2: association of demographic factors and aggression score and Table S3: association of demographic factors and aggression score.

## Data Availability

The data that support the findings of this study are openly available in Mendeley Data at https://doi.org/10.17632/8wn96r2btp.1.
