# Supplementary material for: Owner‐Directed Canine Aggression in Thailand: Triggers, Associated Factors and Clinical Interventions
Source: Vet Med Int. 2026 Jul 31;2026:6224941. doi: 10.1155/vmi/6224941 (PMC13426483; doi:10.1155/vmi/6224941)
Supplement: Supplementary file 1 — Supporting Information Table S1: demographic characteristics, Table S2: association of demographic factors and aggression score and Table S3: association of demographic factors and aggression score. [file VMI-2026-6224941-s001.docx]

**Supplementary data**

**Table S1** Demographic characteristics (N = 80).

| **Owner Characteristics** | **Number** | **Percentage** |
| --- | --- | --- |
| **Gender** |  |  |
| Man | 13 | 16.25 |
| Woman | 67 | 83.75 |
| **Age (year)** |  |  |
| 20-30 | 9 | 11.25 |
| 31-40 | 21 | 26.25 |
| 41-50 | 25 | 31.25 |
| 51-60 | 18 | 22.50 |
| > 60 | 7 | 8.75 |
| **Experienced dog owner (year)** |  |  |
| ≤ 5 | 32 | 40.00 |
| > 5 | 48 | 80.00 |
| **Dog characteristics** | **Number** | **Percentage** |
| **Sex** |  |  |
| male | 51 | 63.75 |
| female | 29 | 36.25 |
| **Breed** |  |  |
| Mix | 23 | 28.75 |
| Pure | 57 | 71.25 |
| **Age (year)** |  |  |
| 1-4 | 57 | 71.25 |
| 4-8 | 19 | 23.75 |
| >9 | 4 | 5.00 |
| **Reproductive status** |  |  |
| Neuter | 45 | 56.25 |
| Intact | 35 | 43.75 |
| **Dog size** |  |  |
| Small-to-Medium (< 20 kg) | 36 | 45.00 |
| Medium-to-Large (10-25 kg) | 44 | 55.00 |
| **Environmental characteristics** | **Number** | **Percentage** |
| **House** |  |  |
| undetached house | 74 | 92.50 |
| detached house/condo | 6 | 7.50 |
| **Living space** |  |  |
| cage | 8 | 10.00 |
| indoor | 48 | 60.00 |
| semi-indoor | 24 | 30.00 |
| **Chained** |  |  |
| yes/often | 21 | 26.25 |
| No/rare | 59 | 73.75 |
| **Number of humans in the household (>18 years)** |  |  |
| 1 | 1 | 1.25 |
| 2-4 | 55 | 68.75 |
| >4 | 20 | 25.00 |
| **The presence of kids in the household**  **(age < 18 years old)** |  |  |
| Yes | 20 | 25.00 |
| No | 60 | 75.00 |
| **Number of dogs in the household** |  |  |
| 1 | 39 | 48.75 |
| 2 | 20 | 25.00 |
| >2 | 21 | 26.25 |
| **The presence of other animals** |  |  |
| yes | 24 | 30.00 |
| no | 56 | 70.00 |
| **Duration (months)** |  |  |
| <12 | 23 | 28.75 |
| 13-24 | 15 | 18.75 |
| >24 | 42 | 52.50 |
| **Management** | **Number** | **Percentage** |
| **Food** |  |  |
| Commercial | 59 | 73.75 |
| Homemade | 21 | 26.25 |
| **Number of meals** |  |  |
| ≥ 3 times/day /all day | 16 | 20.00 |
| 1 time/day | 10 | 12.50 |
| Twice a day | 54 | 67.50 |
| **Body condition score (1-9; 1=thinnest, 9=fattest)** |  |  |
| 1-3 (Underweight) | 10 | 12.50 |
| 4-6 (Optimal) | 49 | 61.25 |
| 7-9 (Overweight) | 21 | 26.25 |
| **Concurrent chronic disease** |  |  |
| Yes | 16 | 20.00 |
| No | 64 | 80.00 |
| **Duration for walking (min)** |  |  |
| None | 13 | 16.25 |
| <15 | 26 | 32.50 |
| 15-30 | 31 | 38.75 |
| 30-60 | 10 | 12.50 |
| **Human-dog interaction** | **Number** | **Percentage** |
| **Positive reinforcement** |  |  |
| Never/very rare | 3 | 3.75 |
| Often | 77 | 96.25 |
| **Punishment-based training** |  |  |
| Never/very rare | 51 | 63.75 |
| Often | 29 | 36.25 |
| **Hugging** |  |  |
| Never/very rare | 12 | 15.00 |
| Often | 68 | 85.00 |
| **Head petting** |  |  |
| Never/very rare | 12 | 15.00 |
| Often | 68 | 85.00 |
| **Body petting** |  |  |
| Never/very rare | 8 | 10.00 |
| Often | 72 | 90.00 |
| **Dog kissing** |  |  |
| Never/very rare | 15 | 18.75 |
| Often | 65 | 81.25 |
| **Walk a dog** |  |  |
| Never/very rare | 29 | 36.25 |
| Often | 51 | 63.75 |
| **Traveling with a dog** |  |  |
| Never/very rare | 42 | 52.50 |
| Often | 38 | 47.50 |
| **Play with toy** |  |  |
| Never/very rare | 24 | 30.00 |
| Often | 56 | 70.00 |
| **Artificial bone** |  |  |
| Never/very rare | 42 | 52.50 |
| Often | 38 | 47.50 |
| **Scent** |  |  |
| Never/very rare | 65 | 81.25 |
| Often | 15 | 18.75 |
| **Listen Music** |  |  |
| Never/very rare | 55 | 68.75 |
| Often | 25 | 31.25 |
| **Watch TV** |  |  |
| Never/very rare | 43 | 53.75 |
| Often | 37 | 46.25 |
| **Dog training to follow command** |  |  |
| Never/very rare | 31 | 38.75 |
| Often | 49 | 61.25 |
| **Sleeping behavior** |  |  |
| Usually sleep in the same room as the owner | 39 | 48.75 |
| Not sleeping with the owner | 41 | 51.25 |
| **The amount of time the dog is left alone at home daily**  **(without anyone present)** |  |  |
| > 9 | 44 | 55.00 |
| 6-9 | 9 | 11.25 |
| 3-6 | 15 | 18.75 |
| 1-3 | 12 | 15.00 |

**Table S2** Association of demographic factors and aggression score analyzed using Mann-Whiteney U test (univariable analysis).

| **Factors** | **Statistics (U)** | **P value** | **Effect size (r)** |
| --- | --- | --- | --- |
| Owner gender | 395.00 | 0.60 | 0.09 |
| Owner experience | 654.50 | 0.26 | -0.14 |
| Presence of kid | 507.00 | 0.30 | -0.15 |
| Pure/mix breed | 516.50 | 0.13 | -0.21 |
| Dog sex | 681.50 | 0.56 | -0.07 |
| Dog size | 665.00 | 0.21 | -0.16 |
| Chronic disease | 413.00 | 0.23 | -0.19 |
| Neuter status, Y/N | 744.00 | 0.67 | 0.05 |
| Positive training | 103.50 | 0.76 | 0.1 |
| Leashing | 489.50 | 0.15 | 0.2 |
| Punish | 612.00 | 0.20 | 0.17 |
| Hug | 305.00 | 0.16 | -0.25 |
| Head pet | 247.00 | 0.03* | -0.39 |
| Body pet | 222.00 | 0.29 | -0.22 |
| Dog kiss | 446.00 | 0.61 | -0.08 |
| Walk a dog | 733.00 | 0.95 | 0.01 |
| Travel | 573.00 | 0.03* | 0.28 |
| Play with toy | 550.50 | 0.20 | -0.18 |
| Artificial bone | 597.00 | 0.05 | -0.25 |
| Scent | 378.50 | 0.17 | -0.22 |
| Music | 665.00 | 0.81 | 0.03 |
| TV | 691.50 | 0.31 | -0.13 |
| Training | 694.00 | 0.51 | -0.08 |
| Company when eat | 249.50 | 0.54 | 0.13 |
| Sleep with owner | 790.00 | 0.93 | 0.01 |
| House type | 154.50 | 0.21 | -0.30 |
| Food type | 448.00 | 0.06 | -0.27 |

Each factor contained 2 group comparisons. *Represent statistically significant difference, p < 0.05.

**Table S3** Association of demographic factors and aggression score analyzed using Kurskal Wallis test (univariable analysis)

| **Factor** | **χ²** | **df** | **P value** | **Effect size (ε²)** |
| --- | --- | --- | --- | --- |
| Owner age | 7.06 | 4 | 0.13 | 0.09 |
| No. of human | 1.46 | 2 | 0.48 | 0.02 |
| Dog age | 2.67 | 2 | 0.26 | 0.03 |
| Period of fostering | 1.58 | 2 | 0.45 | < 0.01 |
| Body score | 1.04 | 2 | 0.59 | 0.01 |
| Living place | 4.54 | 2 | 0.10 | 0.05 |
| Duration of walk | 4.89 | 3 | 0.17 | 0.06 |
| Food type | 5.83 | 2 | 0.05 | 0.07 |
| Mealtime | 1.90 | 2 | 0.38 | 0.02 |

Each factor contained 3-5 group comparison. No statistically significant was observed (p ≥ 0.05)
